# Tweets from the forest: using Twitter to increase student engagement in an undergraduate field biology course

**DOI:** 10.12688/f1000research.6272.1

**Published:** 2015-03-31

**Authors:** Lauren Soluk, Christopher M. Buddle

**Affiliations:** 1 Department of Integrated Studies in Education, Faculty of Education, McGill University, Montreal, QC, H3A 1Y2, Canada; 2 Centre for Teaching and Learning, Mohawk College, Hamilton, ON, L9C 1E9, Canada; 3 Department of Natural Resource Sciences, McGill University, Macdonald Campus, Ste-Anne-de-Bellevue, QC, H9X 3V9, Canada

**Keywords:** Twitter, active learning, medium theory, communication

## Abstract

Twitter is a cold medium that allows users to deliver content-rich but small packets of information to other users, and provides an opportunity for active and collaborative communication. In an education setting, this social media tool has potential to increase active learning opportunities, and increase student engagement with course content. The effects of Twitter on learning dynamics was tested in a field biology course offered by a large Canadian University: 29 students agreed to take part in the Twitter project and quantitative and qualitative data were collected, including survey data from 18 students. Students published 200% more public Tweets than what was required, and interacted frequently with the instructor and teaching assistant, their peers, and users external to the course. Almost 80% of students stated that Twitter increased opportunities for among-group communication, and 94% of students felt this kind of collaborative communication was beneficial to their learning. Although students did not think they would use Twitter after the course was over, 77% of the students still felt it was a good learning tool, and 67% of students felt Twitter had a positive impact on how they engaged with course content. These results suggest social media tools such as Twitter can help achieve active and collaborative learning in higher education.

## Introduction

Social media tools have infiltrated teaching practices across many educational settings and a body of research shows that the tools can increase engagement (Junco *et al.*, 2010; Luttrell, 2012; McCool, 2011). However, understanding how social media may directly influence learning dynamics remains understudied, and more detailed empirical studies are required. This is especially important as more social media tools are developed and gain popularity, research needs to be redirected to investigate the impacts of specific media across the full spectrum of teaching and learning.

Twitter, a prevalent social media tool, has become a major player in education. While some skepticism remains by some teaching faculties, there are many who recognize the perceived benefits of using Twitter in the classroom, including the following:

- Restricting posted content to 140 characters forces student to clarify thoughts into concise information bullets. Bergtrom (as cited in Tinti-Kane, 2013) shares that integrating Twitter is not used for the sole purpose of social connecting but “the 140-character strength forces student to gather their thoughts and state clearly a hypothesis or a conclusion” (p. 1).
- Short posts encourage discussion. While Twitter is asynchronous by nature, the short posts can happen in live time and can facilitate a synchronous-like conversation. These conversations can happen both in and out of class between students, teaching faculty and others interested in the topic, increasing communication ( Hedge, 2012). Hedge (2012) explains that posting tweets is similar to passing notes between students, which results in a more cohesive community.
- Twitter has the ability to authenticate course material. When information is posted to the online Twitter forum, outside members have the ability to interact with the posted content. This interaction can validate student work ( Hawks, 2012; Hedge, 2012). Students are then accountable to the public and not just the professor.
- Twitter has the ability to archive digitally all course content if students use a course hashtag (#). Using the hashtag, students have on-demand access to relevant course material ( Hawks, 2012; Hedge, 2012).
- Darling *et al.* (2013) explain that Twitter can help establish connections to like-minded researchers, subsequently helping students to evolve ideas into actual scientific output. From this perspective, students can transition from student to researcher. This provides validation for the student’s work.

These insights provide a foundation to understand the possible positive effects of Twitter in an educational setting. However, there must be a clear recognition that the tool itself will influence the messages sent by students, and received by others. This relates to McLuhan’s (1964) thinking about how the medium through which content is delivered can impact the way the message and content is received and understood by the receiver, or, “the medium is the message” (McLuhan, 1994, p. 7). The stimulation of different senses through the medium dictates the way in which the message and content is received, making the medium and the message inseparable. McLuhan’s (1964) medium theory also suggests that a medium has the ability to change between hot and cold.

Hot media deliver large packets of content in high definition that fill the recipient with information ( McLuhan, 1964). Since hot media offer a high quantity of content, the recipient requires low levels of participation and interaction with the content to construct understanding. The learning acquisition metaphor (AM) is a learning theory that helps to explain and support McLuhan’s understanding of hot media. The acquisition metaphor explains that learning can occur by direct content transmission from teacher to student. This type of direct content transmission reduces the levels of student participation ( Sfard, 1998). Active learning principles suggest that for students to make meaning of the content, students must actively engage with the content in different ways. Since hot media deliver larger packets of information, students do not need to actively construct meaning of the content; all information and meaning is provided through the medium. An example of this is a traditional post-secondary lecture ( McLuhan, 1964), which may be associated with lower levels of interaction and engagement.

Cold media, in contrast, send small amounts of information thereby requiring recipients to “fill in the blanks” in order to make meaning of the content ( McLuhan, 1964, p. 22). This requires a high level of active participation and interaction with the content. Active learning pedagogy suggests that when learners have autonomy and ownership over content through active participation, material and content will be better understood ( Jonassen & Land, 2012). Sfard (1998) explains that this type of learning can be characterized using a participation metaphor (PM). The PM emphasizes a shift in the role of the learner. The learner in the PM is actively engaged with the content and other students to construct their own knowledge structures ( Sfard, 1998). This is considered to be effective pedagogical practice.

This change between hot and cold media is dependent on the context in which the recipient engages with the material, suggesting the personal motivations of a student, for example, can impact their participation level, thereby influencing the medium. We posit that Twitter is a cold medium, since Twitter restricts content to a small amount of content and it follows that the recipients (i.e., students) are required to engage actively with the content through high levels of participation to make meaning of it. The medium, Twitter, would be directly responsible for shaping the message.

 We sought to directly quantify the effects of Twitter on learning dynamics in an undergraduate field biology classroom, and to place our findings in the context of how the medium shapes the message, and on the medium type. Our first objective was to test the effects of the medium, Twitter, on student engagement, and our second objective was to demonstrate how Twitter affected communication patterns and interactions within the course parameters.

## Methods

### Course setting

A field biology course at a large Canadian post-secondary institution was the selected research site as part of the course was designed around student-led, group-based research projects about natural history ( Ernst *et al.*, 2014). For about a one-month period of the course, and over several intense four-hour laboratory periods, groups of student worked together on a research project about a species occurring within a forest setting. As part of these projects, students were required to write about their research projects using various tools, including Twitter. To study the effects of Twitter on undergraduate learning dynamics, it was essential that the course had a defined and well-articulated learning outcome that could be associated with Twitter. In the field biology course, the fourth learning outcome was concerned with science communication: “ **(iv)** Communicate about Natural History & Ecology to general and specialized audiences (including the instructor), through a range of media (e.g., social media, written works, oral presentations),” thus ensuring that Twitter integration into the course design was both purposeful and meaningful. To achieve this, students were provided an outreach strategy, using Twitter, worth 15%, in the course syllabus. The outreach strategy stated:

**Outreach (15%) –** during November, groups will be publishing blog posts about their selected study species and their research project. The post is to include an overview of the biology and natural history of the study species, outline of research project, personal anecdotes related to the projects, links to other resources and scientific literature, and photos or other media. This is worth 5% as each group member shall receive the same grade on the blog post. Throughout the term, groups will also use Twitter as a means to disseminate information about their study species, and as a means to network and collaborate about natural history within and beyond the course boundaries. Early in the term, the groups will make an Twitter account for their project, and will be required to tweet at least 4 times within a 24 hour time period of each field laboratory (i.e., including those outside of the research project laboratories). Tweets will be ‘relevant’ to [course]. At least 10 tweets are to be released within 48 hours of each group’s blog post being published. Grading will be based on quality of tweets, evidence of communication within the course, and with people beyond the boundaries of the course. This is worth 10% and each group member shall receive the same grade.

To maximize the potential for student success, students were given a “How-to” Twitter guide, the assignment details and a document containing reasons for using Twitter in the course.

Within the Twitter assignment details, it was emphasized that Twitter was engaging, collaborative, and provided networking capabilities. It was explained that Twitter enables tracking and archiving of content, has academic and teaching value, and provides validation (i.e. it gives students an audience other than the professor and teaching assistant). There were explicit instructions and expectations, and student were given the following grading rubric as related to Twitter:

1. Quantity and format: groups met the minimum requirements for tweets, hashtags used appropriately.
2. Etiquette and Professionalism: tweets and interactions on Twitter were mature, professional and written with appropriate tone.
3. Engagement: evidence of collaboration, networking and engagement, both within the course and beyond; use of replies, direct tweets, and interactions.
4. Quality: content of tweets of high quality, relevant to study species, research projects and the overall course content.
5. Links and Photos: appropriate use of linked content in tweets, including photos (e.g., study).

### Twitter data collection

The purpose of our project was to specifically assess how the use of Twitter in the field biology course affected learning dynamics, and as such, the tweeting process, and tweets themselves, became our quantitative data. Data about the students’ tweets, collected using various methods including, observations, tweet analysis, and individual student interviews, were conducted by the first author. First, pending consent, students were asked to provide a copy of their Twitter profile statistics, which were obtained through Twitter. Each groups’ tweets were analyzed in two distinct ways: content (i.e., the body of the tweet) and the tweet interactivity levels (i.e., number of tweets, tweets to and from different users) in order to provide understanding of how students shaped content to fit within the Twitter restrictions and interaction levels. All participants were asked to complete a survey to gain insight into their thoughts around Twitter use; 18 students (out of the 29 who consented to the survey, out of the 43 registered in the class) participated in the survey. The complete survey can be found in Appendix A. Field observations were also conducted throughout during the laboratory periods which provided insights into the different levels of Twitter users in each group. Subsequently, two members from each group were asked to participate in interviews (one high level Twitter user and one low level Twitter user). Combined, a total of five users participated in the interviews (three active users, two low-level users). The qualitative interview results were used to supplement the quantitative data from the tweets, and the quantitative data from the survey results.

## Results and discussion

**Figure 1.**
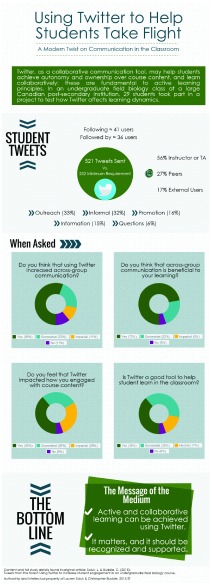
Infographic to highlights general findings on the use of Twitter to increase student engagement in an undergraduate field biology class. Data from surveying 18 of 29 consenting students (see Appendix A for full survey details and Appendix B for raw data from survey).

**Figure 2.**
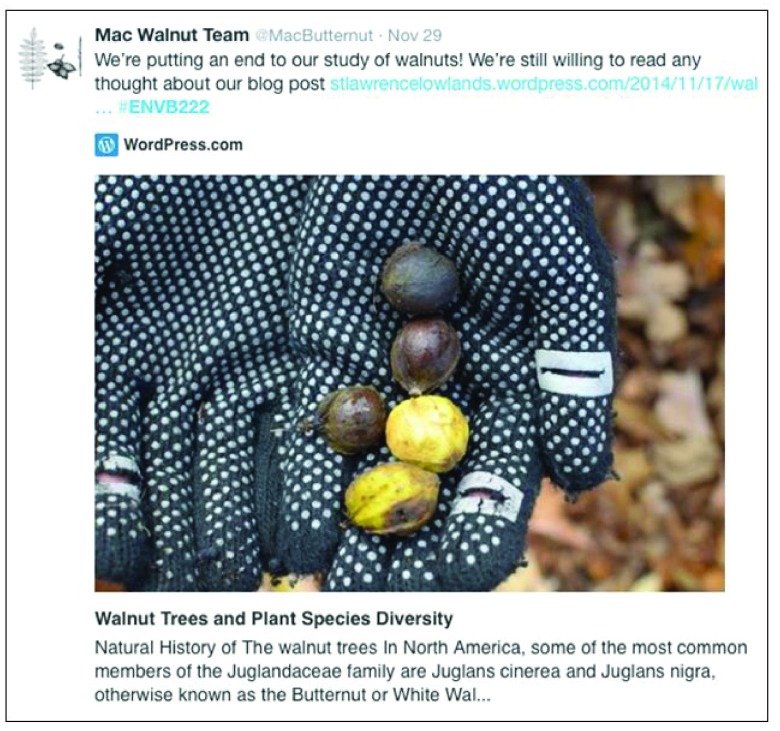
Example of a “Promotional” Tweet published from one of the student groups. In this Tweet, the student are promoting a link to their own published blog post.

**Figure 3.**
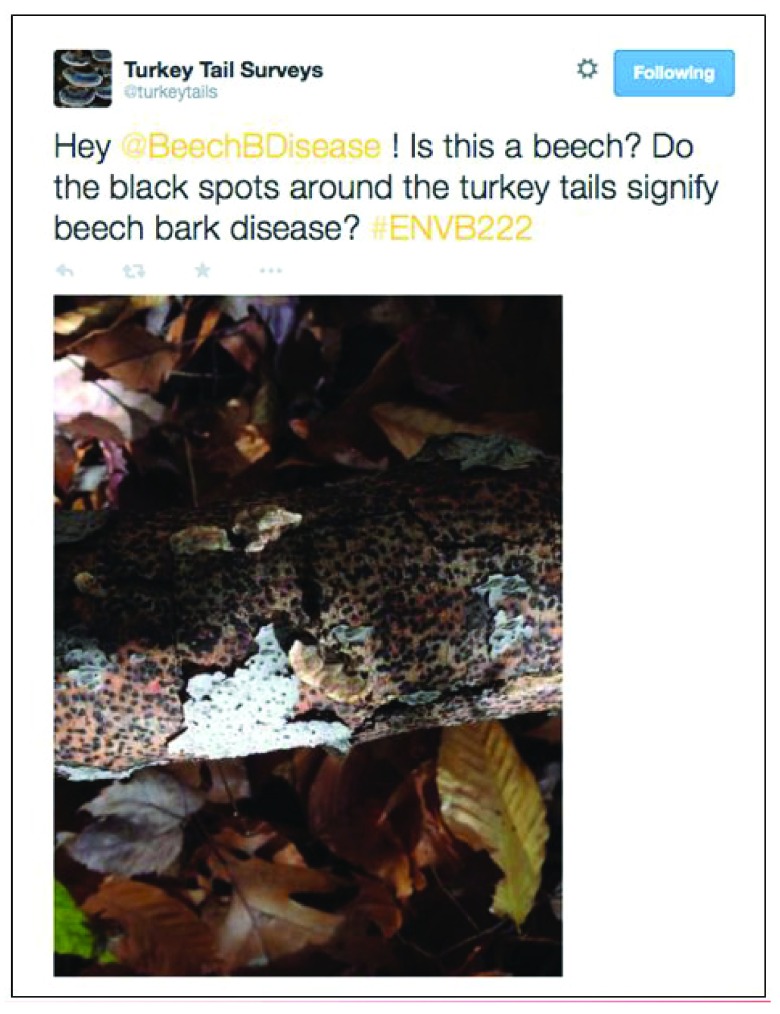
Example of an “Informal Communication” Tweet published from one of the student groups. In this Tweet, one group of students is asking a question to another student group, about something they observed during a field laboratory.

**Figure 4.**
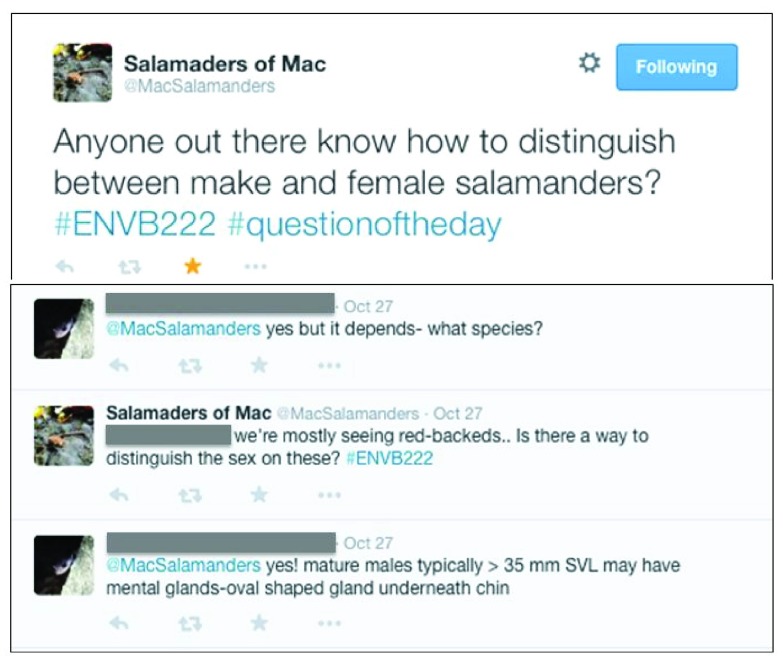
Example of a “Question” Tweet published from one student group. In this example, a Twitter user, external to the course and from a different country, interacted directly with the students, in response to their question.

Based purely on the number of tweets sent by our four study groups, students clearly used Twitter, sending over 200% more tweets than minimally required ( Figure 1). Of these, less than 1% of the tweets were text only, meaning that almost all tweets were content-rich, including a link, photograph, other user, or hashtag. Student groups were able to gather followers, and connect directly with other users ( Figure 1), and they used Twitter for various means. Example tweets illustrate how students used Twitter to promote their own writing about their project ( Figure 2), ask questions of other groups ( Figure 3), or ask questions of experts from outside of the classroom, to troubleshoot in the field ( Figure 4).

Active learning pedagogy states that when students are actively involved in the learning process, it can result in enhanced memory recall and learning ( Cherney, 2008; Craik & Lockhart, 1972). Two important features of active learning are manipulation of content by students through discussion and hands-on activity, and second, student collaboration and cooperation ( American Psychological Association, 1997).

The general results ( Figure 1) illustrate how Twitter promoted active learning: the medium allowed students to actively engage with other learners, collaborate in a hands-on manner, cooperate among each other and with other users, and actively engage with course content. Although the majority of the interactions were between students and other members of their direct learning community ( Figure 1), it is notable that 17% of interactions were with users external to the class: without Twitter these interactions would not have occurred.

Raw data from student survey on the use of Twitter to increase engagement in biology courses Data from the survey are shown. The survey data provide quantitative information from all selected study participants. Click here for additional data file. Copyright: © 2015 Soluk L and Buddle CM 2015 Data associated with the article are available under the terms of the Creative Commons Zero "No rights reserved" data waiver (CC0 1.0 Public domain dedication).

## Medium is the message

Given that Twitter is only a medium, in order to engage with it, students had to choose which content to communicate through the channel. The only constraints that students encountered were the content instructions (i.e., content had to be related to course material) and the 140-character restriction. Together, the limited restrictions gave students control of how to use Twitter (e.g. network building) and control over the content of tweets. Given that the students could control content, we categorized tweets *post hoc*, and were able to separate the 521 tweets into the following distinct functions, in descending order of frequency: outreach (i.e. tweets to interact with users, 33%), informal communication (i.e. general purpose tweets or status updates, 30.7%), promotion (i.e. trying to promote a topic, idea, and/or link, 15.9%, e.g., Figure 2), information (i.e. tweets with informational and/or educational content, 14.5% of tweets), and question (i.e. users asking for help and/or guidance, 5.7%, e.g., Figure 4). The content and text structure of each tweet changed as Twitter was used for different purposes suggesting that the medium has various uses in a classroom context.

The 140-character restriction provided a limitation on tweet content. The students did not all view the 140-character favourably and found it frustrating. For example, Andrew (All names were given pseudonyms to protect student identity) explained,

*Yeah, and many times I felt like well, come on I want this word in but how can I, you know, cause I didn’t want to shorten words cause it was still for the class. So I was trying to find a way to rephrase, and you’d rephrase that and because you rephrased it the second sentence would make sense. So you’d have to, it was much harder grammatically than like, that I expected.* (Andrew)

Despite this, the students indicated the restriction had an impact on their thinking: the limitation helped students clarify thoughts, as they had to consciously think about how to structure the text and content of the tweet. This idea is supported by Emily, as she stated,

*It’s hard to say because like since it’s limited it also gets people to write like meaningful things, like straight to the point instead of just dragging on sentences and stuff.* (Emily)

This restructuring of tweet content is a characteristic of active learning as the students actively manipulated the content to fit within the character restriction of a tweet.

 Given that 83% of the students had never used Twitter, the newness of the tool shaped the impact of the medium as the students had to learn how to use it. Despite this, the students evaluated Twitter and its associated benefits and drawbacks. There were few negative judgments of Twitter. For example, even a low-level Twitter user was able to see the potential benefits of using Twitter:

*So it’s interesting because despite you saying that you don’t use Twitter you still have a very positive view of it?* (Lauren)
*Yeah I know really because I kind of like, I don’t know how to say... because I kind of think about Facebook and I know how it’s like a great, because I just don’t know Twitter enough. But maybe if I knew Twitter how I know Facebook and if I used Twitter how I, how I used Facebook I know it would really like I would really like it. And I would really like students to go on there and to share things, and to, but it’s just the social media [the professor] chose I’m not really comfortable with. But I know it’s a great, a great idea to use social media to learn because, well Facebook I would have been really more comfortable but it’s okay.* (Kristen)

Even though many students found that Twitter was a useful tool in the classroom, a large number of participants indicated that they would not continue to use it after the class finished: 67% of respondents said they would no longer continue to use Twitter outside of the classroom, where 28% said they would (one student said “maybe”) ( Appendix B). These results were unexpected as we assumed that if students have a favourable reaction to Twitter, they would continue to use it in additional settings. Simply put, they may just prefer other tools for their personal use. Since Twitter was limited to the classroom context, picking the tool, as an instructor, is extremely important as it has a direct impact on student engagement and potential successful interactions with course content. The medium must serve a direct purpose, which is outlined by a learning outcome. Moreover, students’ recognition of the value of such tools is critical as it provides them with understanding of how specific tools might be potentially useful in their future endeavours.

## Interactivity and communication

Since Twitter was used in a group setting, it was anticipated that the medium would have an effect on group dynamics, intra-class dynamics, and communication with outsiders. Given that the Twitter responsibilities were associated with a particular group, not individuals, the integration of Twitter had strong implications for relations within groups, as the students had to identify individual Twitter roles. This design was purposeful as it was a strong belief of the second author (based on previous experience) that not all students would gravitate towards the medium; the survey results and field observations supported this belief.

The interviews and field notes suggested that there was one group member who was often responsible for the majority of tweets and other group members’ involvement ranged from non-participation to active involvement with Twitter, suggesting the emergence of a Twitter hierarchy, yet despite the varying levels of Twitter use, 78% of participants still felt that they contributed to creating tweets. Some interviewees indicated that they participated through idea sharing and research:

*Twitter was not my, I didn’t fully use Twitter. I posted one picture one time and some, some little comments, but most of the time I was like, I found some, some articles on the Internet. And I was like I tell them on Facebook I sent them, and [Marie] was like okay I’m gonna post this. And name was more like the, the poster and I, and I kind of did a little research.* (Kristen)
*Most of the tweets I participate in is when we were doing the data collection in the-, so it was like on the moment I would say, “Oh, you should tweet that, it’s fun,” or something like at.* (Carla)

Despite the different levels of Twitter use, the excerpts also suggest that the students still worked together to form their tweets. The data provided supports the claims by the second author as there were different levels of Twitter users. It is reasonable to suggest that the group format directly contributed to the successful integration of Twitter as it provided those who favoured Twitter the opportunity to use the medium and those who were less inclined to use the medium and alternative way of contributing to the work without penalization.

In the laboratory setting, the research groups were dispersed across a large forested reserve. Without communication devices, groups would have had limited contact with each other and integrating Twitter into the course design provided students with a tool to connect with peer groups and outsiders. The survey data demonstrates that Twitter provided student groups with a unique opportunity to interact with each other and the instructor in an online setting ( Figure 1), and students generally believe that this across-group communication is beneficial to their own learning ( Figure 1). Qualitative data supported this as the majority of students indicated that across-group dialogue was enhanced by Twitter integration and that increased peer dialogue was beneficial for student learning. For example, one student mentioned that outsider participation resulted in engagement and provided a more interactive student experience.

*Because we really get to see other like in a simple classroom like wouldn’t really know the other team’s research. But on Twitter it’s public and everyone can see it, and like everybody that’s not in the class also can add their own little thing to do it. I don’t know it’s more engaging and interactive.* (Kristen)

Twitter provided the students with an opportunity to interact and communicate with anonymous outsiders. To use Twitter effectively, students had to build publicly accessible networks and to do so required active participation from students. The students had to actively search and follow like-minded users, as it was their responsibility to develop networks for communication. Quantitative data from the individual group Twitter interactivity profiles demonstrated that students had numerous unique connections with outside users and they often had repeat connections with these users ( Figure 1). The students indicated that the interactions and communication with outsiders as well as other groups provided them with the opportunity to interact with individuals to discuss topics associated with their research goals and course content.

*Well yeah I can say [it’s helpful] because some, some people when they were asking us questions we, it gave us another way of thinking about stuff. And, and sometimes they gave us also papers also to prove their point, so it did help me, well us in a team just to have more papers also to look at to answer our questions, so yeah.* (Marie)

Combined, 56% of interactions were with the instructor or teaching assistant, 27% of interactions were with peers groups, and 17% of interactions were with external users ( Figure 1). Through collaboration, students can work together to discuss and manipulate content thereby creating, and participating in, communities of practice ( Wenger, 2000). Such communities help develop and maintain competencies that contribute to learning and identity, all of which align with the characteristics of active learning pedagogy ( Craik & Lockhart, 1972).

## Contributions to engagement

The various findings, including tweet content and creation and interactivity and communication suggest that student levels of engagement were affected by using Twitter. Engagement indicators are based on a variety of criteria, including academic challenge (i.e., higher order thinking, learning strategies, reflective and integrative learning, and quantitative reasoning), learning with peers (i.e., collaborative learning, discussions with diverse others), experiences with faculty (student-faculty interactions and effective teaching practices), and campus environment (quality of interactions and supportive environment) ( National Survey of Student Engagement, 2014, p. 1). Using Twitter in the classroom addressed three of the engagement indicators: students were required to actively collect data and reflect and synthesize this content to share out using Twitter (academic challenge), although not required, students engaged in active peer discussion through the medium, as well as collaborated with outside anonymous users, and finally, there was a high degree of interaction between the instructor/teaching assistants and the students (experiences with faculty). Students, when surveyed, felt that Twitter impacted how they engaged with course content ( Figure 1), which subsequently contributed to generally positive outlooks of Twitter as a learning tool ( Figure 1). Since we suggest that Twitter is a cold medium, its usefulness is finally determined by the students’ willingness to engage with it. Students must actively engage with the cold medium of Twitter and the content to get the full benefit as the tool itself cannot alter teaching and learning but only encourage active participation.

## Implications for Teaching and Learning

Our results have implications for many educators. Twitter is determined by user interactions with the platform and in our work, the results were positive because the majority of students felt that they contributed, directly and indirectly, to the Twitter project. This resulted in increased communication, collaboration, and critical thinking, all of which was mostly related to course content. This demonstrates that students were actively involved in the learning process. A contributing factor to the successful integration of Twitter was its group form given that it allowed for differing levels of individual contribution based on comfort levels without impacting academic performance.

 The results showed that students had different levels of interactions with the medium. This meant that students who enjoyed using the medium could do so, and the students who were not drawn to the medium could have lower participation. Essentially, the group regulation, a by-product of classification, resulted in agreed upon Twitter roles that were comfortable for group members. As a functioning and regulated group they could effectively use Twitter and achieve the intended learning goals.

 If Twitter were integrated into course design as an individual-based project, we speculate that the results would not have been as positive since not all students are drawn to Twitter. This is evident by the differing levels of student interaction with Twitter. Also, Bernstein (1996) suggests that uncontrolled communication can threaten identity and security. Given that is it essential for students to develop networks to facilitate dialogue and collaboration with outsiders, Twitter profiles should remain public. If Twitter profiles were individualized and public, students might not have been likely to approach Twitter since uncontrolled communication can be considered a threat to identity and security. To counter this, if students had private profiles, networks would have been more difficult to establish, thereby reducing the probability and ease of dialogue and communication with outsiders. These factors suggest that to effectively integrate Twitter into course design, Twitter may best be approached as a group task, and a group-based Twitter handle and alias ensure privacy of individual students.

 It is also important to consider the interactivity levels of users for an additional purpose: given that not all students prefer to use the medium, the students must be provided with additional activities to engage with course material. Educators must employ various strategies to engage students; Twitter is only one tool that can facilitate engagement and collaboration.

A key concern for educators is about how to integrate Twitter successfully into pedagogical practice. Educators must establish boundaries to guide student use of Twitter. In this study, students were required to create tweets related to course content. This restriction forced the students to use the medium primarily for educational purposes, thereby facilitating increased engagement directly with course content. In addition, there was a clear grading schema to assess and evaluation of student’s use of Twitter: this provided an important expectation and framework for the use of social media. Given the various interactivity levels with the medium, we would speculate that without a grade attachment, Twitter uptake would not be as successful. Also, given some students’ unfamiliarity with Twitter, providing documents or lessons on Twitter provides students with the tools to be effective Twitter users.

To effectively build and establish networks, Twitter profiles must remain public. This allows for an easy, bi-directional relationship between followers and users. The benefit of network building is increased access to new and alternative learning materials through tweets. Also, with a larger Twitter network, students will have the opportunity to communicate and collaborate with various users. However, this also means that the instructors of teaching assistants should also be familiar with the tools, and leverage their own networks as required.

## Conclusion

The purpose of the study was to investigate the effects of Twitter on undergraduate learning dynamics. To do so, we explored the functionality and purpose of tweets, Twitter’s effect on communication, and the impact of Twitter on engagement. The data analysis revealed that using Twitter:

- required students to learn about Twitter and evaluate its usefulness in a classroom setting,
- facilitated engagement with course content,
- facilitated and enhanced intra-group dialogue,
- promoted and enhanced communication and collaboration with group outsiders, and,
- facilitated active learning with students being responsible for actively manipulating and restricting content to form, and response to, tweets.

Twitter can be effectively used in an undergraduate classroom and can be effectively integrated into a course design. As a result of Twitter use, students actively participated in their learning, which resulted in collaboration, increased communication, and facilitated student engagement. The message of the medium is that active and collaborative learning can be achieved, it matters, and it should be recognized and supported.

## Data availability

The data referenced by this article are under copyright with the following copyright statement: Copyright: © 2015 Soluk L and Buddle CM

Data associated with the article are available under the terms of the Creative Commons Zero "No rights reserved" data waiver (CC0 1.0 Public domain dedication). 

*Figshare:* Raw data from student survey on the use of Twitter to increase engagement in biology courses. Doi: http://dx.doi.org/10.6084/m9.figshare.1360179 ( Soluk & Buddle, 2015).

Twitter data were analyzed in the following manner: first, participant groups provide a copy of their Twitter user profile; these profiles included every Twitter interaction that occurred during the semester, including interactions with users, tweet content, and date and time stamps. This information was into a larger document and analyzed tweet functions. Tracking included: overall profile statistics, including the number of followers, tweets sent from mobile and stationary devices, and the number of picture and video tweets, number of user interactions with the professor, teaching assistant, peer groups, and anonymous users. After this analysis was complete, this information was set aside and incorporated with other data at a later date.

Second, individual interview transcripts were analyzed. In this process, each interview and coded the data based on ‘what was happening’; the categories were descriptive of actions and events; each category had a rule of inclusion.

Finally, the survey results were integrated into the thematic analysis to provide a level of dimensionality to the findings. While interviews lend insight into the students’ consciousness, the survey data provided quantitative information from all selected study participants. It helped to provide an overview of student feelings. These results provided a general understanding of student perspective.

All data was integrated under the themes established from the student interviews, thus resulting in the final product. Raw data from the survey is accessible through this article.
