# Supplementary material for: Tweets from the forest: using Twitter to increase student engagement in an undergraduate field biology course
Source: F1000Res. 2015 Mar 31;4:82. [Version 1] doi: 10.12688/f1000research.6272.1 (PMC4648182; doi:10.12688/f1000research.6272.1)
Supplement: Supplementary file 2 [file f1000research-4-6728-s0001.tgz › 44fb59ee-7fb0-433e-801e-c401926cd4f8.pdf]

Appendix A: Student Survey Tool

Dear Student,

I am asking that you complete a survey about your Twitter use in the ENVB 222 course. The collected results will provide insight into student perceptions of Twitter use in the classroom. The results will supplement the additional research I have gathered. It will help me to understand the effects of Twitter on learning dynamics. If for any reason you are not comfortable answering a question(s), you are able to skip over the question(s).

SurveyMonkey is an online survey tool. SurveyMonkey will collect you IP address. Please be aware that your IP address will not be collected in the survey results. All results from the survey will be stored in a protected electronic format to which only my supervisor and myself will have access too. All identifiable collected data from the survey will be password protected. Further, the data will be stored on a personal computer that will be password-protected. Please be aware that while all security measures will be taken, any information sent over the internet may be intercepted by a third party. Do I have you consent to continue?

- Yes
- No

Participant Name: ________________________________ (Please print)

Participant E-Mail: ________________________________

Date: ________________________________

General

1. Which gender do you identify with: Male Female

2. Please select your age group:

18 - 23	24 - 30	30 +

Social Media Practices

These questions refer to you social media practices BEFORE participating in this class.

3. Prior to this class beginning, how often did you use the following social media applications for personal reasons? Please select the most applicable box in each category.

	Several times daily	Once a day	2-6 times a week	Once a week	2-3 times a month	Once a month	Never
Facebook							
Twitter							
Google+							
Linkedin							
Skype							
Blog							

4. Prior to this class beginning, overall, how important was social media in your personal life? Please select the box that most applies to you.

Very important	Somewhat important	Neither important or unimportant	Somewhat unimportant	Very unimportant

5. Prior to this class beginning, how often did you use the following social media applications for school reasons? Please select the most applicable box in each category.

	Several times daily	Once a day	2-5 times a week	Once a week	1-3 times a month	Once a month	Never
Facebook							
Twitter							
Google+							
Linkedin							
Skype							
Blog							

6. Prior to this class beginning, overall, how important was social media in your school life? Please select the box that most applies to you.

Very important	Somewhat important	Neither important or unimportant	Somewhat unimportant	Very unimportant

Twitter Focus

7. At the start of the class (when the Twitter component was first introduced), how did you feel about using Twitter in ENVB 222? Please select all that apply.

Angry	Fearful	Stressful	Overwhelmed	Impartial	Content	Optimistic	Enthusiastic	Empowered

8. Prior to this class beginning, did you have a personal Twitter account: Yes No

(Survey participants who selected yes to the previous question were asked to complete questions 9 – 15. If they selected no, they were re-directed to question 16).

9. How long have you had a Twitter account? Please circle the most applicable response:

1 week	1 month	6 months	1 year	Over a year

10. How often do you Tweet? Please circle the most applicable response:

Several times daily	Once a day	2-5 times a week	Once a week	1-3 times a month	Once a month	A few times a year	Once a year

11. Do you Retweet (RT) or reply?	Yes	No
12. Do you Direct Message (DM)?	Yes	No
13. Do you use hashtags (#)?	Yes	No
14. Do you post pictures?	Yes	No
15. Do you post links to additional webpages?	Yes	No

Post-Class Twitter Perceptions

16. Prior to this class beginning, have you ever used Twitter in a classroom setting?
- Yes
- No

17. Professor posted a "How to" Twitter guide online. How much of the document did you read?
- The whole thing
- Approximately half
- I didn't read it

18. Did the document help you to understand and use Twitter?
- Yes
- Impartial
- No

19. How often did you log onto your group's Twitter account?
- Only on Tuesday
- Daily
- 3-5 times per week
- 1-2 times per week
- Bi-weekly
- Once per month
- Never

20. How often did you Tweet?
- Only on Tuesdays (minimum requirements of 4 tweets within the 24 hours time slot)
- Daily
- 3-5 times per week
- 1-2 times per week
- Bi-weekly
- Once per month
- Never

21. You may or may not have been directly involved with the Tweeting process. Do you still feel like you contributed to your group's tweets?
- Yes
- No

22. Do you feel that using Twitter impacted how you engaged with the course content?
- Yes
- Somewhat
- Impartial
- No

23. Did you find the 140-character length helped you to clarify your thoughts when creating your tweets?
- Yes
- Somewhat
- Impartial
- This doesn't apply to me

24. Do you think that across-group communication (i.e. increased dialogue between groups) is beneficial to your learning?
- Yes
- Somewhat
- Impartial
- No

25. Do you think that using Twitter increased across-group communication?
- Yes
- Somewhat
- Impartial
- No

26. Overall, do you think Twitter is a good tool to help students learn in the classroom?
- Yes
- Somewhat
- Impartial
- No

27. Do you think you will continue to use Twitter outside of this classroom experience?

- Yes
- No
