# Supplementary material for: Tweets from the forest: using Twitter to increase student engagement in an undergraduate field biology course
Source: F1000Res. 2015 Mar 31;4:82. [Version 1] doi: 10.12688/f1000research.6272.1 (PMC4648182; doi:10.12688/f1000research.6272.1)
Supplement: Supplementary file 3 [file f1000research-4-6728-s0002.tgz › 4d548dc4-e28d-4951-95a0-b41fdafc1397.pdf]

Appendix B: Raw Data

1.

Which gender do you identify with?

**Answer Options**	**Response Percent**	**Response Count**
Male	27.8%	5
Female	72.2%	13
	answered question	**18**
	skipped question	**0**

2.

Please select your age category:

**Answer Options**	**Response Percent**	**Response Count**
18 - 23 years	94.4%	17
24 - 30 years	5.6%	1
30 + years	0.0%	0
	answered question	**18**
	skipped question	**0**

3.

Prior to this class beginning, how often did you use the Prior to this class beginning, how often did you use the following social media applications for personal? Please select the most applicable box in each category.

**Answer Options**	**Several times daily**	**Once a day**	**2-6 times a week**	**Once a week**	**2-3 times a month**	**Once a month**	**Never**	**Response Count**
Facebook	11	3	1	0	0	2	1	18
Twitter	0	0	1	0	1	1	15	18
Google+	0	1	1	0	0	1	15	18
Linkedin	0	0	0	0	1	0	17	18
Skype	1	1	0	3	2	4	7	18
Blog	0	1	0	1	1	1	14	18
							answered question	**18**
							skipped question	**0**

4.

Prior to this class beginning, overall, how important was social media in your personal life? Please select the box that most applies to you.

**Answer Options**	**Response Percent**	**Response Count**
Very important	5.6%	1
Somewhat important	66.7%	12
Neither important or unimportant	11.1%	2
Somewhat unimportant	5.6%	1
Very unimportant	11.1%	2
	answered question	**18**
	skipped question	**0**

5. **Prior to this class beginning, how often did you use the Prior to this class beginning, how often did you use the following social media applications for school reasons? Please select the most applicable box in each category.**

Answer Options	Several times daily	Once a day	2-6 times a week	Once a week	2-3 times a month	Once a month	Never	Response Count
Facebook	0	2	4	2	2	4	4	18
Twitter	0	0	0	0	0	0	18	18
Google+	0	0	2	0	1	1	14	18
Linkedin	0	0	0	0	0	0	18	18
Skype	0	0	0	0	1	3	14	18
Blog	0	0	0	0	0	1	17	18
							answered question	**18**
							skipped question	**0**

6. **Prior to this class beginning, overall, how important was social media in your school life? Please select the box that most applies to you.**

Answer Options	Response Percent	Response Count
Very important	0.0%	0
Somewhat important	38.9%	7
Neither important or unimportant	22.2%	4
Somewhat unimportant	11.1%	2
Very unimportant	27.8%	5
	answered question	**18**
	skipped question	**0**

7. **At the start of the class (when the Twitter component was first introduced), how did you feel about using Twitter in [the course]? Please select all that apply.**

Answer Options	Response Percent	Response Count
Angry	5.6%	1
Fearful	5.6%	1
Stressful	16.7%	3
Overwhelmed	27.8%	5
Impartial	50.0%	9
Content	11.1%	2
Optimistic	38.9%	7
Enthusiastic	22.2%	4
Empowered	0.0%	0
	answered question	**18**
	skipped question	**0**

8. **Prior to this class beginning, did you have a personal Twitter account?**

Answer Options	Response Percent	Response Count
Yes	22.2%	4
No	77.8%	14
	answered question	**18**
	skipped question	**0**

9.	**How long have you had a Twitter account? Please select the box that most applies to you.**

**Answer Options**	**Response Percent**	**Response Count**
1 week	0.0%	0
1 month	0.0%	0
6 months	0.0%	0
1 year	25.0%	1
More then 1 year	75.0%	3
	answered question	**4**
	skipped question	**14**

10.	**How often did you Tweet? Please select the most applicable.**

**Answer Options**	**Response Percent**	**Response Count**
Several times daily	0.0%	0
Once a day	0.0%	0
2-5 times a week	0.0%	0
Once a week	0.0%	0
1-3 times a month	0.0%	0
Once a month	50.0%	2
A few times a year	0.0%	0
Once a year	50.0%	2
	answered question	**4**
	skipped question	**14**

11.	**Do you Retweet (RT) or reply?**

**Answer Options**	**Response Percent**	**Response Count**
Yes	50.0%	2
No	50.0%	2
	answered question	**4**
	skipped question	**14**

12.	**Do you Direct Message (DM)?**

**Answer Options**	**Response Percent**	**Response Count**
Yes	0.0%	0
No	100.0%	4
	answered question	**4**
	skipped question	**14**

13.

**Do you use hashtags (#)?**		
**Answer Options**	**Response Percent**	**Response Count**
Yes	50.0%	2
No	50.0%	2
	answered question	**4**
	skipped question	**14**

14.

**Do you post pictures in your Tweets?**		
**Answer Options**	**Response Percent**	**Response Count**
Yes	50.0%	2
No	50.0%	2
	answered question	**4**
	skipped question	**14**

15.

**Do you post links to additional webpages in your Tweets?**		
**Answer Options**	**Response Percent**	**Response Count**
Yes	0.0%	0
No	100.0%	4
	answered question	**4**
	skipped question	**14**

16.

**Prior to this class beginning, have you ever used Twitter in a classroom setting?**		
**Answer Options**	**Response Percent**	**Response Count**
Yes	5.6%	1
No	94.4%	17
	answered question	**18**
	skipped question	**0**

17.

**Professor posted a "How to" Twitter guide online. How much of the document did you read?**		
**Answer Options**	**Response Percent**	**Response Count**
The whole thing	27.8%	5
Approximately half	55.6%	10
I didn't read it	16.7%	3
	answered question	**18**
	skipped question	**0**

18.

Did the document ("How To" guide) help you to understand and use Twitter?

Answer Options	Response Percent	Response Count
Yes	50.0%	9
Impartial	50.0%	9
No	0.0%	0
	answered question	**18**
	skipped question	**0**

19.

How often did you log onto your group's Twitter account?

Answer Options	Response Percent	Response Count
Only on Tuesday	0.0%	0
Daily	22.2%	4
3-5 times per week	38.9%	7
1-2 times per week	11.1%	2
Bi-weekly	11.1%	2
Once per month	5.6%	1
Never	11.1%	2
	answered question	**18**
	skipped question	**0**

20.

How often did you Tweet?

Answer Options	Response Percent	Response Count
Only on Tuesdays (minimum requirements of 4 tweets within the 24 hours time slot)	5.6%	1
Daily	5.6%	1
3-5 times per week	22.2%	4
1-2 times per week	38.9%	7
Bi-weekly	11.1%	2
Once per month	5.6%	1
Never	11.1%	2
	answered question	**18**
	skipped question	**0**

21.

You may or may not have been directly involved with the Tweeting process. Do you still feel like you contributed to your group's tweets?

Answer Options	Response Percent	Response Count
Yes	77.8%	14
No	22.2%	4
	answered question	**18**
	skipped question	**0**

22. **Do you feel that using Twitter impacted how you engaged with the course content?**

Answer Options	Response Percent	Response Count
Yes	38.9%	7
Somewhat	27.8%	5
Impartial	27.8%	5
No	5.6%	1
	answered question	**18**
	skipped question	**0**

23. **Did you find the 140-character length helped you to clarify your thoughts when creating your tweets?**

Answer Options	Response Percent	Response Count
Yes	11.1%	2
Somewhat	33.3%	6
Impartial	44.4%	8
This doesn’t apply to me	11.1%	2
	answered question	**18**
	skipped question	**0**

24. **Do you think that across-group communication (i.e. increased dialogue between groups) is beneficial to your learning?**

Answer Options	Response Percent	Response Count
Yes	72.2%	13
Somewhat	22.2%	4
Impartial	0.0%	0
No	5.6%	1
	answered question	**18**
	skipped question	**0**

25. **Do you think that using Twitter increased across-group communication?**

Answer Options	Response Percent	Response Count
Yes	55.6%	10
Somewhat	22.2%	4
Impartial	11.1%	2
No	11.1%	2
	answered question	**18**
	skipped question	**0**

26.

Overall, do you think Twitter is a good tool to help students learn in the classroom?

Answer Options	Response Percent	Response Count
Yes	38.9%	7
Somewhat	38.9%	7
Impartial	16.7%	3
No	5.6%	1
	answered question	**18**
	skipped question	**0**

27.

Do you think you will continue to use Twitter outside of this classroom experience?

Answer Options	Response Percent	Response Count
Yes	29.4%	5
No	70.6%	12
Other (please specify)		2
	answered question	**17**
	skipped question	**1**
