# Supplementary material for: Tweets from the forest: using Twitter to increase student engagement in an undergraduate field biology course
Source: F1000Res. 2015 Mar 31;4:82. [Version 1] doi: 10.12688/f1000research.6272.1 (PMC4648182; doi:10.12688/f1000research.6272.1)
Supplement: Peer review history

# Peer review: Referee response for version 1

**DOI:** 10.5256/f1000research.6728.r8893

**Published:** 2015-07-07

**Authors:** Gavan P. L. Watson

**Affiliations:** 1 Teaching Support Centre, University of Western Ontario, London, ON, Canada

As the uses of social media in post-secondary instruction continue to proliferate across across disciplines, engaging in research that describe classroom approaches and measures outcomes is a valuable addition to this area of scholarship. I'm happy to approve of this paper for: 1) its contributions to the scholarship of practice related to using Twitter in the classroom and 2) its methodology to measure the tool's impact. Innovative, is the pedagogical design of a group-level use of the tool. The strengths of the research begin with the strong link and alignment between the course-level learning outcomes and the use of Twitter. This finding, and the implications of the importance of choosing e-Learning tools aligned with student learning, are important contributions beyond the field of teaching and learning with social media. 

Broadly, I would encourage the authors to make explicit links to the teaching and learning literature in the opening of the paper to strengthen the underpinning pedagogical arguments. For example, there are also more recent (and compelling) references than McLuhan in the literature to support the claim that large-class lectures are more passive. They write that active student engagement is "…considered to be effective pedagogical practice"—again, a link to research that substantiates this claim would improve the argument being laid out.

While it may be implicit, I assume that students' use of Twitter *on smartphones* was integral to the tool's positive impact on engagement across the forested reserve. In this case, it is not *just* Twitter that had an impact, rather it was the combination of the platform (Twitter) and the internet-connected mobile device. This is a broad consideration for future research: how does the mobile, ubiquitous nature of smartphones, used in conjunction with a tool such as Twitter, impact student engagement and outcomes?

I have read this submission. I believe that I have an appropriate level of expertise to confirm that it is of an acceptable scientific standard.

# Peer review: Referee response for version 1

**DOI:** 10.5256/f1000research.6728.r8181

**Published:** 2015-05-11

**Authors:** Joshua Drew

**Affiliations:** 1 Department of Ecology, Evolution and Environmental Biology, Columbia University, New York, NY, USA

While many people on twitter sing its praises for use in class, I think there is a real lack of worked examples of how *exactly* one would use the medium. Moreover, cases that have demonstrated impacts are precious few. This piece addresses both of these shortcomings with a convincing and even handed approach to how one could use technology to augment an undergraduate field based class. They show that it works well for a given period of time, but that like all tools, there are appropriate and inappropriate uses for twitter. Having used the tool in several of my cases, I can wholeheartedly agree with the lack of engagement post-class - the medium simply isn't for everyone. However I think the authors here clearly show how using Twitter can help a class expand beyond the traditional boundaries and help students learn to engage in informal science discourse.

I have read this submission. I believe that I have an appropriate level of expertise to confirm that it is of an acceptable scientific standard.
